# Technology for Automated Production of High-Performance Building Compounds for 3D Printing

**DOI:** 10.3390/ma17153829

**Published:** 2024-08-02

**Authors:** Adam Hutyra, Magdalena Bańkosz, Bożena Tyliszczak

**Affiliations:** 1 Department of Materials Engineering, Faculty of Materials Engineering and Physics, Cracow University of Technology, 37 Jana Pawla II Av., 31-864 Krakow, Poland; adam.hutyra@doktorant.pk.edu.pl; 2 ATMAT Sp. z o.o., Władysława Siwka 17, 31-588 Krakow, Poland

**Keywords:** automation of construction processes, 3D printing in the construction industry, high-performance construction compounds

## Abstract

Three-dimensional printing technology in construction is a rapidly growing field that offers innovative opportunities for design and construction execution. A key component of this process is the automated production of high-performance construction mixtures that meet specific requirements for strength, fluidity, and setting speed. This overview article outlines the history and development of 3D printing technology in the construction industry, describes various printing technologies, and discusses the properties and requirements for construction mixes. Special attention is given to automated systems for batching and mixing ingredients, which increase the precision and efficiency of production. The different types of construction mixes used in 3D printing and the main technical and operational challenges associated with their application are also presented. The article’s conclusions highlight the potential of this technology to revolutionize the construction industry by improving efficiency and reducing costs and project lead times.

## 1. Introduction

Three-dimensional printing technology, also known as additive manufacturing, has revolutionized various industrial sectors, including construction, by offering new possibilities for design and execution. The origins of 3D printing date back to the 1980s, when it was primarily focused on prototyping and producing small objects. However, over time, this technology has evolved and found applications on larger scales, including in construction. In the construction industry, 3D printing technology gained prominence in the early 21st century when experimentation with printing structural and architectural elements began. Innovations in building materials, such as 3D-printed concrete, have enabled the creation of more complex and durable structures [1]. Initially, 3D printing was used to create models and prototypes, but it quickly became a tool for executing complex architectural projects that would be difficult or impossible to achieve using traditional methods [2].

One of the first groundbreaking applications of 3D printing in construction was the creation of experimental structures such as bridges and small buildings. These early projects demonstrated the potential of 3D printing to create custom shapes and forms, paving the way for further innovations. As the technology developed, 3D printing began to be applied on an increasingly larger scale, encompassing entire residential and commercial buildings and even residential complexes. Technological advancements also enabled the development of automated dosing and mixing systems, significantly increasing the efficiency and precision of producing building mixtures. These innovations have made 3D printing in construction popular as a method that allows for waste reduction, shorter construction times, and increased design flexibility [3,4]. The next step in the development of 3D printing in construction was integration with digital design tools such as Building Information Modeling (BIM) software. This combination has enabled more efficient design and planning, as well as the automation of the entire construction process from design to execution [5].

Currently, 3D printing technology in construction continues to develop, with new materials, techniques, and applications being explored worldwide. Researchers and engineers aim to further increase the strength and durability of printed structures, as well as to expand the possibilities of 3D printing to larger projects and more complex applications. This technology promises not only a revolution in the way we build but also in how we think about the spaces we inhabit. Automated production of high-performance building mixtures plays a key role in modern construction, especially in the context of the growing popularity of 3D printing technology. Automation of the production processes of building mixtures brings a number of benefits that significantly impact efficiency, quality, and sustainable development in construction. Foremost is the precision and repeatability offered by automated production. These systems are capable of precisely measuring and mixing ingredients according to strictly defined recipes, minimizing the risk of human error and ensuring the uniformity of the mixture. This consistency is crucial for maintaining high-quality standards in construction projects. Additionally, automated systems can significantly speed up the production process of mixtures, which is invaluable in rapidly developing construction projects. Reducing the time required to prepare the mixture allows for faster progress on the construction site, ultimately leading to a shorter overall project completion time. Automated production of building mixtures also contributes to increased work safety by reducing direct contact of workers with potentially harmful substances and heavy machinery. These systems can be monitored and controlled remotely, reducing the need for direct human intervention and the associated risk of accidents. Another important aspect is the optimization of material usage. Automation allows for precise dosing of ingredients, thereby minimizing waste and contributing to sustainable development. Efficient material management not only lowers costs but also reduces the negative impact of construction on the natural environment. In the context of 3D printing, automated production of high-performance building mixtures is essential for achieving optimal results. Precisely prepared mixtures enable the creation of more complex structures with better accuracy and durability, opening new possibilities for innovative design and construction [6,7].

Moreover, 3D printing technology in construction is gaining importance as an innovative solution with the potential to significantly impact the industry. Despite its substantial contribution to global economies (9% of GDP) and high employment rates (7–8.5% of the workforce), the construction sector struggles with low productivity and limited technology adoption. Total worldwide spending on construction was USD 11.4 trillion in 2018 and is expected to increase to USD 14 trillion by 2025. Although 3D printing in construction is still largely in the research and development phase, it offers benefits such as faster construction, reduced material waste, lower labor demands, and improved worksite safety. However, its widespread implementation faces challenges related to high infrastructure costs and potential impacts on the labor market, especially in countries where construction is a major source of employment. In the future, with further development and technology adaptation, 3D concrete printing could revolutionize the construction industry, but it requires additional research and careful cost analysis [8].

In summary, automated production of high-performance building mixtures is a critical element of modern construction, translating into better quality, greater efficiency, and safety, as well as reduced environmental impact. It forms the foundation for further development and innovation in the industry, especially in the area of construction supported by 3D printing.

## 2. Basics of 3D Printing Technology in Construction

### 2.1. Description of the 3D Printing Process and Its Applications in Construction

Three-dimensional printing technology in construction, also known as additive manufacturing, has significantly transformed the construction industry by enabling the creation of complex structures quickly and cost-effectively [1,6]. This process begins with creating an accurate digital 3D model of a building or structure using computer-aided design (CAD) software. This model is then converted into a series of thin, horizontal layers, allowing the 3D printer to sequentially reproduce the design layer by layer [9,10]. The choice of materials for 3D printing in construction is diverse and depends on the specific requirements of the project, including special types of concrete, resins, and even metals. The 3D printers used in this sector are significantly larger than those known from other applications and can take the form of large industrial robots with movable arms that precisely apply the material according to the design [11,12,13]. The entire printing process can take from a few hours to several days, depending on the size and complexity of the project, and upon completion, the structure often requires additional processing, such as sanding or painting, to improve its appearance and physical properties. Three-dimensional printing has found wide application in construction, from the erection of residential and commercial buildings to the realization of unique architectural constructions that would be difficult to execute using traditional methods, as well as the construction of bridges and other infrastructure elements. This technology also offers opportunities for the restoration and conservation of monuments and the creation of homes tailored to individual needs, opening new perspectives for innovative design and construction [14,15,16].

### 2.2. Review of Various 3D Printing Technologies Used in Construction and Their Applications

Nowadays, 3D printing technologies are gaining importance in many fields, including construction, where they offer innovative approaches to design and construction. Different 3D printing technologies, such as Fused Deposition Modeling (FDM), Selective Laser Sintering (SLS), and Stereolithography (SLA), have their unique applications in construction, offering various benefits depending on project requirements [17,18]. FDM technology involves the gradual deposition of material through an extruding nozzle that moves along programmed paths. This method is widely used due to its simplicity, low cost, and the ability to use various materials such as thermoplastics. The recent literature reports indicate the widespread use of 3D printing technology in the processing of both polymer and composite materials. Furthermore, the concept of 4D printing using smart polymer materials is increasingly emerging. However, in the case of construction applications, the latest technologies primarily utilize various forms of three-dimensional techniques [19,20,21]. FDM is primarily used in construction to create conceptual models, prototypes, and structural elements such as fittings and joints [22]. SLS, on the other hand, uses a laser to sinter powdered materials layer by layer, creating objects with high strength and complexity. This technique is particularly useful in construction for elements requiring special mechanical properties, such as structural components or installation parts. The advantage of SLS is the ability to work with various materials, including metals, which allows for the creation of very durable components. SLA, based on curing photopolymer resins under UV light, allows for achieving high precision and surface quality, making it ideal for creating accurate architectural models and intricate forms [23]. In construction, this technology is primarily used to produce architectural details, moldings, and decorative elements. Each of these 3D printing technologies has its specific applications in construction, enabling the creation of structures that would be difficult or impossible to achieve using traditional methods. Advances in this field open new possibilities for architects and engineers, enabling the realization of more sustainable, efficient, and personalized construction projects [24,25]. Table 1 presents various 3D printing technologies and their applications in the construction sector.

## 3. Properties and Requirements of High-Performance Building Mixtures

**Figure 1:**
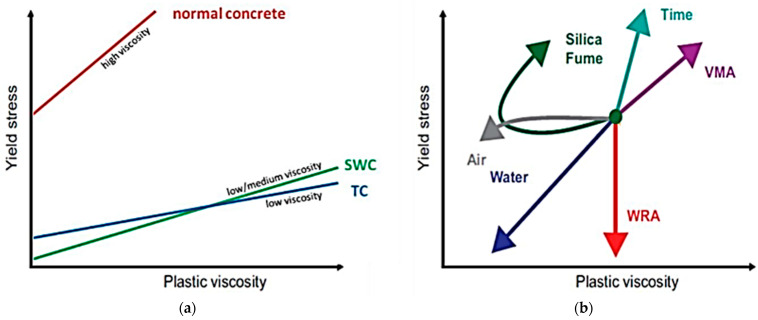
Rheology of an optimized tremie concrete mix in comparison to NC and SCC (**a**), and the influence of different additives and admixtures on the behavior of fresh concrete (**b**) [30].

The properties and requirements of high-performance building mixtures are crucial for achieving the desired structural characteristics and durability. High-performance building mixtures, often characterized as high-performance concrete (HPC) or specialty mortars, must meet rigorous criteria to meet contemporary engineering and architectural demands. The first and fundamental requirement is high compressive strength, which allows structures to withstand significant loads without damage. This strength is achieved through a precisely chosen composition of ingredients, including cement, aggregate, water, and often additives and admixtures that enhance the mixture’s properties. The second key requirement is durability, understood as resistance to external factors such as moisture, temperature changes, chemicals, and corrosion [26]. High durability ensures the longevity of structures and minimizes the need for maintenance and repairs. High-performance mixtures must also exhibit good workability, meaning ease of mixing, transporting, placing, and compacting, while ensuring the desired quality and appearance of the finished structure. This workability is often achieved through the use of superplasticizers, which reduce the amount of water needed without lowering the quality of the mixture. Finally, but equally important, is sustainable development and ecology. High-performance building mixtures increasingly contain recycled materials such as fly ash or slag, contributing to reducing CO_2_ emissions and the use of industrial waste. Analyzing the key properties required for building mixtures for 3D printing is essential to understanding how to optimize materials for this innovative technology. Key properties primarily include rheology, setting time, strength and durability, and compliance with environmental standards. The rheology of building mixtures is crucial as it affects the ability of the 3D printer to precisely apply the material and maintain its shape after application [27,28]. It is worth noting that studies report that the quality of the final product is affected by the rheology of the concrete mixes as well as the speed of printing [29]. Figure 1 shows how various chemical admixtures and additives affect the rheological properties of different concrete mixtures. This chart helps assess how these modifiers interact with the concrete used.

**Figure 2:**
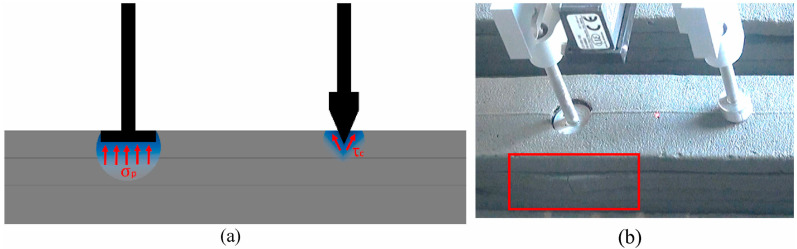
Two-dimensional schematic illustration of compressive and shear stresses in fresh 3D-printed concrete layers during plate and cone penetration (**a**). Plate and concrete penetration tests on real printed layers and associated deformation (**b**) [39].

The mixture must be fluid enough to easily flow through the printer’s nozzle, but at the same time, viscous enough to retain its shape and allow the layers to bond [31,32]. The setting time is another important aspect as it must balance between quick hardening and enough time for application without the risk of collapse or deformation of the layers. Setting too quickly can hinder working with the material, while setting too slowly can threaten the structure’s stability during printing. Strength and durability are critical to ensure that 3D-printed structures can withstand structural loads and environmental conditions over time. Additives such as reinforcing fibers can be included in the mixture to improve its mechanical properties. Compliance with environmental standards is increasingly important in the context of sustainable construction [33,34]. Building mixtures for 3D printing should be developed to minimize their environmental impact, for example, by using renewable or recycled materials. In the context of the growing interest in 3D printing technologies in construction, the key properties of building mixtures have become the subject of intensive research. The development of 3D printing technology, as described in Gibson, Rosen, and Stucker, opens new possibilities for the construction sector, enabling the creation of complex geometries and structures that would be difficult or impossible to achieve using traditional methods. To fully utilize the potential of this technology, building mixtures must meet specific criteria such as appropriate rheology, setting speed, strength, and durability [35]. The publication by Chua and Leong further emphasizes the importance of understanding the principles and applications of 3D printing, which is crucial for developing building mixtures that can be effectively used in this technology. Understanding these principles allows for better adaptation of the mixture’s properties so that they are compatible with 3D printing processes, while ensuring structures of high quality and durability [36]. The study by Akbari and Modarres evaluating the effect of nano-clay and nano-alumina on the fatigue response of bitumen sheds light on the potential benefits of integrating nanomaterials into building mixtures. Although this study focuses on asphalt, the conclusions can be extrapolated to the needs of 3D printing in construction, suggesting that nanomaterials can significantly improve the mechanical properties and durability of building mixtures [37]. Next, in Khan et al., the role of sustainable development in the context of 3D-printed concrete is highlighted. This study points to the need to develop building mixtures that not only meet technical requirements but are also environmentally friendly. Ultimately, the development and optimization of building mixtures for 3D printing require a holistic approach that considers not only technical properties but also aspects of sustainable development and environmental impact [38]. As 3D printing technology advances, it will be important to continue research into new materials and mixing techniques to meet the growing demands of the construction sector. Atta Ur Rehman, Ik-Gyeom Kim, and Jung-Hoon Kim presented research on the development of an integrated sensor–printer instrument that allows for automatic and inline tests of concrete’s penetration, compression, shear, and tensile properties. This system is integrated with a 3D printer and controlled from a central station. This research represents significant progress in characterizing structural build-up and quality control of concrete on-site, showing its potential application in mix design development [39]. An example diagram for this method is presented in Figure 2.

The article by Shayan Ali Khan and colleagues reviews the impact of nanomaterials on the properties of fresh and hardened cement-based materials (CBMs) used in 3D printing. The study focuses on the optimal and maximum dosing of nanomaterials in mixtures, showing that nanomaterials act as thickeners, improving the thixotropic behavior and structural development of 3D-printable CBMs. Additionally, nanomaterials support the hydration process by providing nucleation sites, leading to the refinement of the CBM’s microstructure. Nano silica (NS) stands out for its significant impact on the thixotropy of 3D-printable mixtures, while carbon-based nanomaterials enhance the physico-mechanical properties of 3D-printable CBMs. Nano TiO_2_ (NT) provides self-cleaning capabilities through photocatalysis, and nanocrystalline cellulose (CNC) and nano clay (NC) are mainly used as viscosity-modifying agents in 3D-printable mixtures. This article is a valuable resource for researchers, engineers, and construction professionals looking for information on the latest advances and key challenges in the field of 3D printing for cement-based materials [40]. The most important properties and their assigned requirements for high-performance mixtures are presented in Table 2.

The use of nanotechnology in construction mixtures offers a number of significant benefits that can significantly improve their mechanical and functional properties. Nanoparticles, such as nano silica, can increase the strength and durability of concrete by strengthening its structure at the micro- and nanoscale levels, resulting in better load and corrosion resistance. The effect of nano-additives on improving mechanical properties was demonstrated in [41,42,43]. In addition, nanomaterials can improve the waterproofing and heat resistance of concrete, as well as speed up the setting time of the mix, which optimizes the construction process [44,45,46]. Moreover, a number of studies are also examining other benefits of nano-additives. Many works are concerned with proving that the incorporation of carbon nanotubes into concrete, for example, can provide self-sensing and self-cleaning properties to concrete structures [47,48,49,50].

## 4. Automated Dosing and Mixing Systems

Automated dosing systems play a critical role in modern 3D printing technology in construction, offering the ability to precisely measure the ingredients needed to create a building mixture. Using advanced algorithms and precise sensors, these systems provide exceptional accuracy in measuring individual components such as cement, sand, various additives, and water. The ability to program these systems to follow strictly defined ingredient proportions is a key element that minimizes the risk of human error and ensures the uniformity and consistency of the mixture. Automated dosing enables not only the precise measurement of each component but also the adjustment of mixture proportions depending on the specific requirements of the construction project. This makes the process of preparing the mixture significantly more efficient and less prone to errors, which is essential for achieving optimal results in the 3D printing process [51].

Real-time monitoring systems that detect and correct anomalies during 3D printing in the construction sector are key to ensuring the quality, precision, and safety of printed structures. They use advanced technologies such as force and torque sensors, 3D cameras, and thermographic cameras to monitor the printing process layer by layer. Through image analysis and artificial intelligence algorithms, these systems can immediately detect and correct errors, adjusting printing parameters in real time. Senthilnathan et al. presented a methodology using computer vision (CV) to continuously monitor the printing process and identify defects. Surface texture changes were recorded by extracting texture changes from high-resolution camera images. The methodology used makes it possible to take corrective action while affecting the high quality of products and avoiding the problem of material wastage [52]. On the other hand, Jhun et al. presented an algorithm for calculating comprehensive cross-sectional information including the width, thickness, and area of the extruded concrete filament. The comprehensive results confirm the effectiveness of monitoring the printing process [53].

Additionally, automated dosing systems can be easily integrated with other elements of the production process, allowing for smooth and automated operation from the preparation of the mixture to the printing process. The consistency of the mixture provided by automated dosing has a direct impact on the quality of the final 3D-printed structures. A uniform mixture ensures better adhesion between layers, greater strength, and durability of the printed elements, which is especially important in the context of construction. Moreover, automation of the dosing process contributes to increased work efficiency, the reduction of material waste, and the improved overall economic efficiency of the production process. As a result, automated dosing systems form the foundation for innovative production methods in the construction sector, offering not only improved quality and precision but also contributing to sustainable development through the optimization of material usage and shortening of production time [54,55]. After the precise dosing of ingredients, the next critical stage in the process of preparing a building mixture for 3D printing is automated mixing. Automated mixing systems play a key role in integrating all the ingredients into a uniform, homogeneous mass, which is essential for ensuring the consistency and quality of printed elements. The mixing process is carried out using specially designed mechanical mixers that must be adapted to work with the mixture’s variety, considering its composition, density, and desired final properties. Advanced mixing systems use precise control and monitoring of mixing parameters, such as the rotational speed of the mixers and mixing time, to tailor the process to the specific requirements of a given mixture. This allows for achieving the optimal consistency of the mixture, which is crucial both for the 3D printing process and for the mechanical properties and durability of the final product. Automated mixing ensures the homogeneity of the mixture by thoroughly and evenly distributing the ingredients, including any additives and reinforcements such as fibers or activators [56,57]. This is particularly important for the mixture used in 3D printing, where the uniformity and stability of the mixture have a direct impact on the print quality, including dimensional accuracy, layer adhesion, and surface aesthetics. Moreover, intelligent mixing systems can adapt to changing process conditions, such as temperature or humidity, which can significantly affect the mixture’s properties. These systems can also dynamically adapt to changes in the mixture’s composition, allowing for quick reactions and adjustment of mixing parameters in real-time, which is extremely important in the dynamic construction environment. As a result, automated mixing systems can not only provide high-quality and consistent mixtures but also increase the efficiency of the production process, reducing the time and costs associated with material preparation. Therefore, they are a key component of 3D printing technology in construction, contributing to innovation, improved efficiency, and sustainable development in the construction sector. The integration of automated dosing and mixing systems with 3D printers in construction technology is a key element that ensures the efficiency and continuity of the printing process. This integration allows the process of preparing the building mixture and its delivery to the 3D printer to be automated, which significantly improves the quality and precision of the final constructions [58]. These systems are designed to precisely dose and mix the mixture’s ingredients and then directly deliver the ready material to the 3D printer, eliminating the need for manual material handling and the associated risk of errors or material loss. This integration allows for not only continuous material flow but also ensures that the mixture is delivered to the printer in optimal condition, which is necessary to achieve high print quality. The optimal condition of the mixture means that it has the correct consistency, density, and viscosity, which is critical to ensuring that the material can be precisely applied layer by layer without the risk of collapse or uneven material distribution. Automated dosing and mixing systems are often equipped with advanced sensors and algorithms that monitor and regulate the mixture’s composition in real-time, allowing for the adjustment of its properties to changing printing conditions or specific project requirements. This dynamic adjustment provides high flexibility and adaptability to the process, enabling the production of structures with diverse parameters from a single production line. Integration with the 3D printer also involves the use of management software that coordinates the work of the dosing and mixing systems with the printer’s operation, synchronizing the material delivery rate with the printing speed. This ensures that the entire process, from mixture preparation through to delivery and the printing process, is smooth and automated, minimizing downtime and maximizing production efficiency [59]. The direct integration of automated dosing and mixing systems with 3D printers in construction is a fundamental solution that contributes to the revolution in 3D printing technology, enabling the production of more complex, durable, and precise constructions while optimizing production processes and minimizing material waste. In modern technological systems used in the construction industry, especially in the context of 3D printing, advanced quality control and the ability to adapt to dynamically changing conditions play a crucial role. These systems are equipped with a range of sensors and monitoring systems that enable continuous tracking of key mixture properties, such as viscosity and density. This allows for not only ongoing adjustment of the mixture’s parameters but also a quick response to any changes in external conditions or specific project requirements. Monitoring the mixture’s properties in real-time ensures that its quality is maintained at the highest level throughout the production process. For example, if sensors detect that the mixture’s density is too low or too high, the system can automatically adjust the amount of added components, such as water or cement, to restore the mixture to its optimal state. Similarly, monitoring the mixture’s viscosity ensures that it has the appropriate fluidity, which is crucial for the precise and even application of material layers during the 3D printing process. Automatic quality control plays a key role in ensuring that each batch of the mixture meets the established quality standards. This, in turn, has a direct impact on the durability and strength of the printed structures. By using quality control systems, it is possible to eliminate errors and inconsistencies that could negatively affect the mechanical and aesthetic properties of the printed elements. Adapting to changing conditions is another important aspect of these advanced systems. This can include adapting to changes in ambient temperature and humidity, which can affect the mixture’s setting process, as well as specific project requirements that may require modifications to the mixture’s composition. Thanks to the ability to adapt, these systems can ensure optimal conditions for each stage of the printing process, from mixture preparation to dosing, mixing, and the printing process [58,60].

## 5. Types of Mixtures for 3D Printing in Construction Applications

Three-dimensional printing in construction is gaining popularity due to its ability to rapidly prototype and realize complex structures. An essential aspect of 3D printing technology is the selection of appropriate material mixtures that must meet specific strength, durability, and aesthetic requirements. Below is an overview of various types of mixtures used in 3D printing in the construction sector, based on the latest research.

### 5.1. Concrete Mixtures

Concrete mixtures are the most commonly used in 3D printing in construction. They are adapted for efficient flow through 3D printer nozzles and rapid setting to ensure the structural integrity of printed elements. The study conducted by K Gamage and colleagues focuses on the progress of sustainable 3D concrete printing in Australia, analyzing materials, challenges, and current advancements. Three-dimensional concrete printing (3DCP) is seen as a sustainable and eco-friendly approach to rapid construction, enabling the creation of complex shapes while maintaining high quality and precision [61].

**Figure 3:**
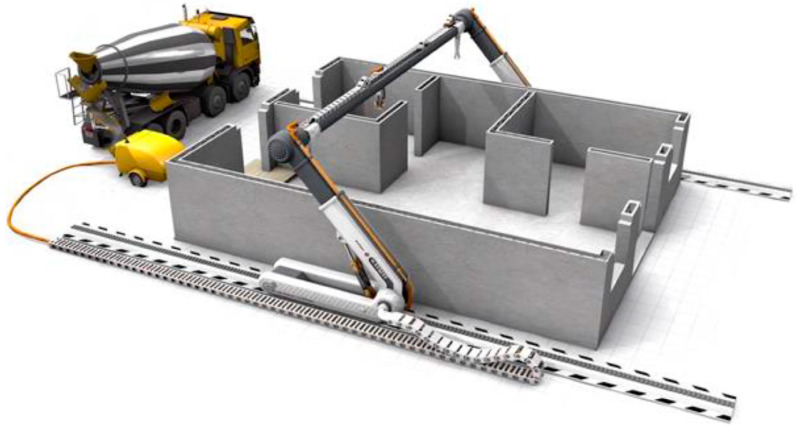
Construction of buildings using 3D concrete printing [62].

The possibility of constructing buildings from concrete mixtures using 3D printing technology is presented in Figure 3 [62]. The automated shotcrete technology, as discussed in the study led by G Isaac and colleagues, presents significant progress in concrete construction, especially in the context of 3D printing. Shotcrete, being a technique of spraying concrete, offers an alternative to traditional construction methods, and its automation opens new possibilities for the construction industry [63]. Unlike conventional 3D printing, which can be limited by nozzle sizes and extrusion speed, shotcrete allows for rapid and efficient application of concrete over large surfaces, significantly increasing the pace of construction. Automating the shotcrete process contributes to sustainability by minimizing material waste and optimizing raw material usage. Precise control of the amount and location of concrete application reduces material excess and increases energy efficiency. Table 3 presents selected properties of concrete mixtures with brief descriptions.

The study conducted by L Zhou and colleagues focuses on analyzing the durability and properties of hardened 3D-printed concrete (3DPC) using bauxite waste as fine aggregate instead of natural sand. The goal of the study was to examine the impact of bauxite waste on the durability and properties of hardened 3D-printed concrete. The research revealed that 3DPC containing bauxite waste exhibits specific characteristics that may affect the material’s durability and hardened properties. Experiments provided a deeper understanding of the interactions between bauxite waste and the cement matrix in 3DPC. The use of bauxite waste as an aggregate in 3D-printed concrete not only contributes to sustainable development by utilizing industrial waste but may also affect the mechanical properties and durability of the concrete [76]. The study conducted by P Narjabadifam and colleagues focuses on the numerical analysis of the seismic behavior of arched roof buildings produced using 3D concrete printing technology (3DPC). The goal of the study was to understand how these structures respond to seismic shocks, which is crucial for buildings located in high-seismic risk areas. The study results can contribute to the development of more sustainable and safer construction practices, especially in the context of seismic construction. Understanding the seismic behavior of 3D-printed buildings is key to their further development and implementation in earthquake-prone areas [77].

Next, recent advancements in self-curing materials for 3D printing in the construction sector highlight the use of photo-responsive and thermo-responsive polymers, photocatalytic cements, shape memory polymers, and geopolymers. Photo-curable resins, which solidify under UV light, are being utilized for precise architectural elements, while thermo-setting polymers, activated by heat, provide high-strength and weather-resistant structures. Photocatalytic cements, incorporating additives such as titanium dioxide, accelerate curing under sunlight and offer self-cleaning properties. Shape memory polymers adapt to environmental changes, enabling the creation of dynamic, responsive structures. Additionally, geopolymers, which cure through thermal activation, offer eco-friendly, high-strength alternatives with chemical resistance [78,79,80,81,82].

### 5.2. Gypsum-Based Mixtures

In recent years, there has been a significant increase in interest in using gypsum in 3D printing technology, particularly in the production of decorative elements, architectural models, and the restoration of monuments. Gypsum, valued for its ease of processing, smooth surface, and rapid hardening, has proven to be an ideal material for precision applications in 3D printing. In one study conducted by O Ahi and colleagues, the focus was on automating extrusion flow control in 3D printing using cement, considering various cement mixtures. This study highlighted the importance of optimizing the flow of extruded material for the quality of the final print, affecting the strength, durability, and aesthetics of the produced elements. Precise adjustment of the amount of extruded material, depending on project requirements and object geometry, is essential for ensuring uniform layers and proper surface quality [83]. Another publication by G Skripkiūnas and colleagues examined the impact of the shape and concentration of cement particles on the rheological properties of cement slurry. The results indicated that the Bingham model, often used to describe the flow of cement slurries, does not accurately reflect reality, as the flow becomes more complex with increasing shear stress, indicating a phenomenon of dilatancy, or increased viscosity of the material with increasing shear stresses. This study highlights the importance of understanding the impact of particle shape and concentration on the technological properties of cement, which directly affects its consistency, fluidity, and pumpability [84]. N Chernysheva, S Shatalova, and V Lesovik focused on analyzing the deformation properties of dense and foamed mortars based on cement and gypsum, considering their application in 3D printing. These studies provide insight into how these mixtures can influence the development of modern construction technologies and 3D printing, considering their mechanical and structural properties. Adding gypsum to cement mixtures significantly affects their properties, improving plasticity, which facilitates processing and slows the hardening process, providing more time for working with the material [85]. G. Bumanis and his team, in a study published in the “*Journal of Composites Science*”, focused on creating a lightweight composite of gypsum and expanded polystyrene granules with 3D printing in mind. This innovative mixture, by reducing weight and improving thermal insulation, can be used not only in construction but also in the production of stage props or architectural models, where lightness and ease of forming are important [86].

**Figure 4:**
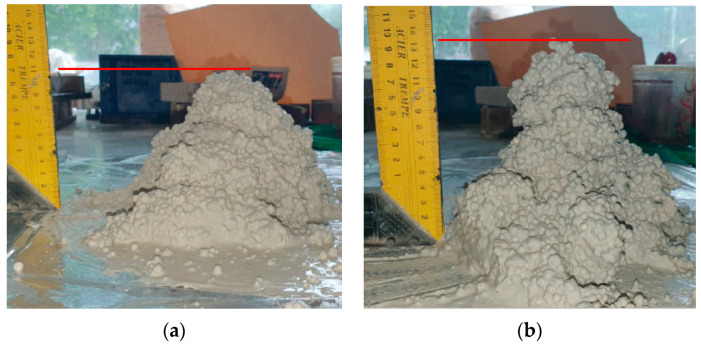
Consistency of gypsum–EPS mixtures with different W/G ratios (**a**) Mixture Ref-1 with W/G = 0.6; (**b**) Mixture Ref-2 with W/G = 0.5 [86].

An example of the consistency of concrete mixtures with varying water-to-gypsum (W/G) ratios is presented in Figure 4. A study conducted by Charai and colleagues focused on lightweight gypsum composites enriched with waste such as fly ash from coal and plant fibers. The goal was to create an ecological material that not only reduces waste but also improves the thermal and humidity comfort of buildings. The study results showed that such additives can significantly improve the insulation properties of gypsum, which is crucial for energy efficiency and user comfort. These studies shed light on the potential of gypsum and its composites in 3D printing, opening new possibilities for innovation in construction and design, both technologically and ecologically [87].

### 5.3. Geopolymers

Geopolymers offer an ecological alternative to traditional concrete mixtures, providing excellent resistance to fire and chemicals. Comparisons between geocement and Portland cement are presented in Table 4.

In a study conducted by ME Perales-Santillan and colleagues, the rheological behavior of metakaolin and lime-based geopolymers was evaluated, indicating their potential in 3D printing applications [89]. The study conducted by Khan and colleagues focuses on understanding the impact of nozzle diameter and printing speed on the construction process of geopolymer concrete structures using 3D printing technology. These two parameters have a significant effect on “buildability”—a term referring to the material’s ability to retain a specific shape during printing and curing. “Buildability” determines how well structures are formed and how durable they are after printing, which is closely related to their subsequent strength and structural stability. The study thoroughly analyzed geopolymer concrete, which is gaining popularity as an ecological alternative to traditional concrete due to its lower carbon emissions and the use of industrial waste. Researchers conducted an experimental analysis of structures printed with this material, considering various process conditions related to 3D printing technology [90]. The study conducted by Chen and colleagues focuses on evaluating the impact of raw materials on the performance of geopolymer systems used in 3D printing technology. The authors discuss the importance of selecting appropriate renewable and low-carbon raw materials both for environmental reasons and in the context of increasing energy efficiency and sustainable resource use in construction. They emphasize that, unlike traditional building materials such as cement products, which generate significant amounts of CO_2_ during production and use, 3D-based geopolymers offer an alternative approach. Geopolymers use renewable raw materials and are characterized by low carbon emissions. Examples of such raw materials include fly ash, slag, or materials from recycled concrete, contributing to a closed-loop economy in the construction industry. Chen and colleagues analyzed the process properties of geopolymers, such as fluidity and thixotropy, which are crucial for the efficiency of the 3D printing process. The microstructure of geopolymers is also studied to better understand how the initial content of silicon, aluminum, and calcium in the silicate raw materials affects the gel structure of geopolymers and microstructural development [91]. In the work of Růžek and colleagues, geopolymers are thoroughly discussed in terms of their applications in both civil and military areas. The authors emphasize that geopolymers, being a type of alkali-activated concrete, have many advantages compared to traditional Portland cement-based materials, including better mechanical and thermal properties, higher chemical resistance, and lower carbon emissions during production. The advantages of geopolymers, such as their resistance to high temperatures and fire, make them an ideal material for applications requiring resistance to extreme conditions, for example, as passive fire protection or in constructions where high-temperature resistance is required. For these reasons, geopolymers can be considered an alternative material in civil construction where such properties are desirable. Interestingly, geopolymers are also a promising material for military applications, where their exceptional explosion resistance can be used to protect infrastructure and vehicles from the effects of explosions. The authors point out that empirical studies and applications of geopolymers by the US armed forces and military research institutes indicate a wide range of potential applications that can utilize their advanced properties. This publication sheds light on the growing role of geopolymers not only as a construction material but also as a potential “ink” for 3D printers. Such 3D printing applications using geopolymers can offer new ways of building structures with complex geometries that were previously difficult or impossible to achieve using traditional construction methods [92]. The study conducted by Lu and colleagues focuses on understanding the impact of adding additives to geopolymers, called retarders, on their rheological properties, which are extremely important in the context of 3D printing construction. Rheological properties, such as shear strength and plastic viscosity, are crucial for maintaining the material’s shape during the creation process and after its completion, and managing these properties through the use of retarders can drastically affect the quality of 3D printing. In this study, researchers developed geopolymers based on fly ash and ultra-fine industrial iron tailings (coal fly ash/superfine iron tailings), creating a material that exhibits the desired properties for use in 3D printing. The additives in the studied geopolymer included, among others, sodium citrates, which act as retarders. The study showed that adding retarders effectively influenced the heat release dynamics during the binding process, which directly impacts the strength interaction and shape stability in the early stages of material strength. The authors also examined the impact of retarders on the geopolymer’s microstructure, discovering that retarders cause a certain portion of the fly ash and iron tailings to remain unused in the reaction, leading to a reduction in early compressive strength compared to the geopolymer without retarders. This was determined through hydration kinetics analysis, where retarders effectively inhibited both the nucleation of crystallization and their growth and diffusion. The work of Lu and colleagues presents a significant achievement in understanding the early retardation mechanism, which allows for improving the 3D printing process of geopolymers and can lead to the wide application of these materials in construction. They provide a significant alternative to traditional building materials, reducing not only environmental impact but also contributing to technological progress and innovation in the construction sector.

**Figure 5:**
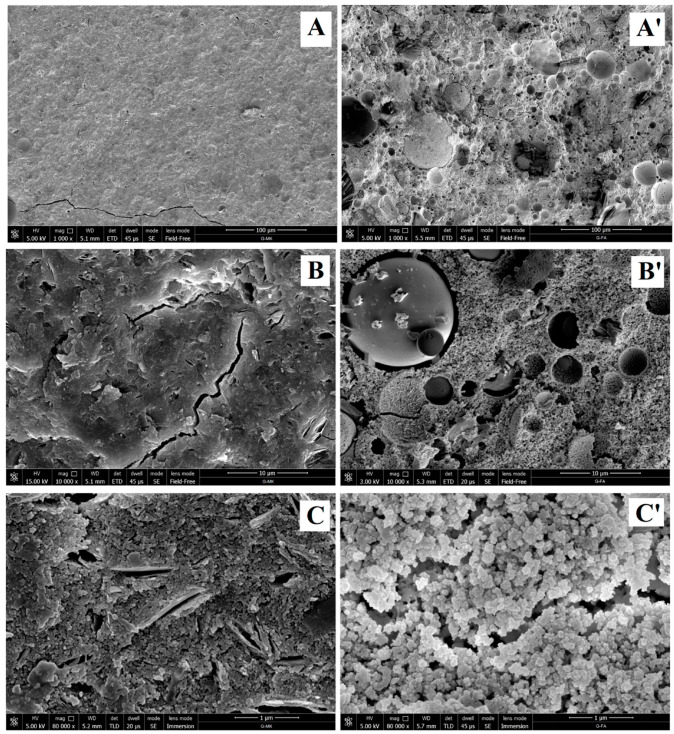
Scanning electron microscope (SEM) micrographs of a metakaolin-based geopolymer (**A**–**C**) and a fly ash-based geopolymer (**A’**–**C’**) [93].

Figure 5 shows the different morphology of geopolymer materials made from different raw materials, i.e., metacaloin and fly ash [93]. Geopolymers have a significant impact on the development of 3D printing technology in construction and other application areas, offering potential benefits in the form of sustainable resource use, improved material characteristics, and expanded civil and military applications. They offer alternative solutions to traditional building materials such as cement, contributing to reducing environmental impact through lower carbon emissions and lower energy consumption [94].

### 5.4. Composites

Composite mixtures, such as concrete reinforced with glass or carbon fibers, are gaining popularity due to their increased strength and durability. In 3D printing technology in construction, the use of composite mixtures offers new possibilities for design and construction realization. These composites, enriched with glass, carbon, or other types of reinforcements, are characterized by significantly better mechanical properties compared to traditional building materials [95]. Using different types of fibers in composites for 3D printing in construction has its own unique advantages and disadvantages. Choosing the right type of fiber depends on specific construction requirements, cost, and environmental conditions. Table 5 presents a summary of selected fibers and their advantages and disadvantages.

Glass Fiber Reinforced Polymer (GFRP), known as GFRP, is an innovative composite material that combines traditional concrete components with the addition of glass fibers [96,97]. This unique mixture significantly improves the mechanical properties of concrete, especially in terms of tensile and bending strength. Glass fibers, being a key element of this composite, are extremely lightweight, which helps reduce the overall weight of the construction while increasing its structural strength. One of the main advantages of GFRP is its corrosion resistance, making it an ideal choice for applications in chemically aggressive environments or where high humidity is present, such as in coastal construction or hydraulic infrastructure [98]. Glass fibers do not corrode like traditional steel reinforcement, significantly extending the lifespan of the structure and reducing the need for maintenance or repairs. Additionally, GFRP is characterized by high strength, surpassing traditional construction materials in tensile and bending tests. This exceptional feature makes the composite suitable for realizing structural elements requiring high strength, as well as in places where precise dimensional tolerances must be maintained. The use of GFRP in 3D printing technology in construction opens new design possibilities, enabling the creation of more complex shapes and structures that would be difficult to achieve using traditional construction methods [99,100]. Three-dimensional printing with glass fiber-reinforced concrete allows precise placement of the material only where needed, minimizing waste and optimizing material usage. In the context of sustainable development, GFRP also offers environmental benefits by increasing the durability of structures and reducing the need for frequent repairs or material replacements, which translates into lower raw material consumption and reduced construction waste [101,102].

**Figure 6:**
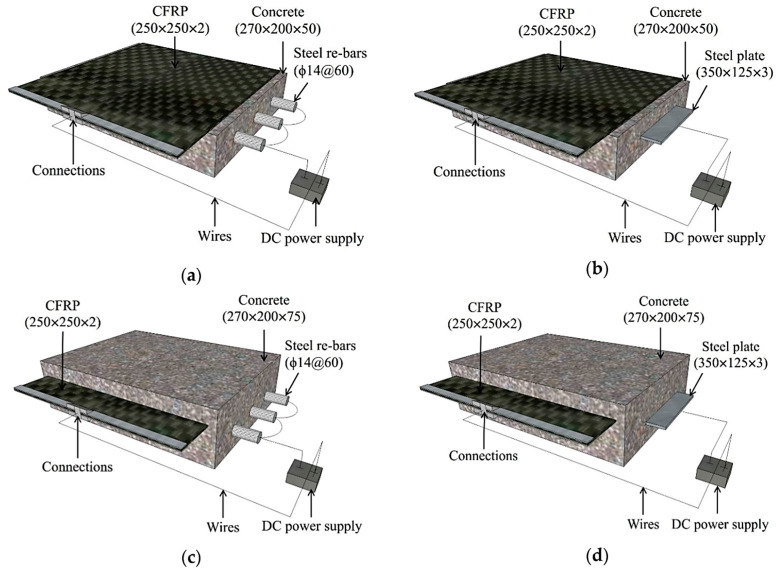
Schematic diagrams of the chloride-contaminated concrete test specimens with bonded carbon fiber-reinforced polymer (CFRP) and embedded steel (all dimensions are in mm): (**a**) Steel rebars—externally bonded CFRP (B-EB specimens); (**b**) Steel plate—externally bonded CFRP (P-EB specimens); (**c**) Steel rebars—internally bonded CFRP (B-IB specimens); (**d**) Steel plate—internally bonded CFRP (P-IB specimens) [105].

Carbon Fiber Reinforced Polymer (CFRP), known as CFRP, is an advanced composite material that uses carbon fiber reinforcement to significantly improve the mechanical properties of traditional concrete. Carbon fibers, being the core of this composite, are characterized by exceptional tensile strength and very low thermal expansion, which translates into significant increases in stiffness and structural strength while reducing the weight of the structure [103,104]. Figure 6 below shows an example of a carbon fiber-reinforced composite material.

The use of CFRP in construction brings a revolution in the way we think about concrete structures. Thanks to its unique properties, carbon fibers allow for the realization of high-strength structures while reducing the thickness of elements, which has a direct impact on lowering the total weight of the structure. This is particularly important in the case of large engineering objects such as bridges, viaducts, or high-rise buildings, where weight reduction can bring significant benefits both in terms of transport and assembly costs, as well as in the context of loads acting on the foundations. CFRP is also distinguished by low thermal expansion, meaning that this material maintains its dimensions over a wide temperature range. This property is extremely valuable in environments where structures are exposed to significant temperature fluctuations, as it minimizes the risk of cracks and deformations caused by thermal changes. In addition to mechanical strength and thermal stability, CFRP also offers excellent resistance to corrosion and chemicals, extending the lifespan of structures and reducing the need for frequent repairs or maintenance [106,107,108]. This makes CFRP an ideal material for applications in harsh environmental conditions, where traditional materials such as steel can quickly degrade. In the context of 3D printing, the use of CFRP opens new possibilities for structural design. This technology allows the precise placement of carbon fibers in specific locations and orientations within the element, maximizing their reinforcing properties where most needed. This enables the creation of structures with complex geometries and optimized mechanical properties that would be difficult to achieve using traditional construction methods [109,110].

Basalt Fiber Composites represent an innovative category of reinforced materials gaining popularity in various engineering fields, especially in construction and composite technology. Basalt fibers, produced from natural volcanic basalt rock, offer a range of unique properties that make them an attractive alternative to traditional reinforcement materials such as glass or carbon fibers [111]. The physical properties of BFs are presented in Table 6.

One of the key advantages of basalt fibers is their exceptional resistance to chemical substances and high temperatures. This property makes basalt composites an ideal choice for applications in extreme environmental conditions where other materials may quickly degrade. Basalt fibers retain their strength and structural integrity even at very high temperatures, making them an excellent choice for thermal insulation, fire protection, and applications in the aerospace and space industries. Furthermore, basalt composites exhibit significant resistance to corrosion, which is particularly beneficial in coastal construction, water infrastructure, and other environments exposed to moisture and salt. This corrosion resistance translates into longer-lasting structures and reduces the need for frequent repairs and maintenance, leading to significant cost savings over the long term [113,114]. Basalt fibers also offer excellent mechanical strength, including tensile and bending strength, comparable to or even surpassing some types of glass fibers. This strength, combined with the lightweight nature of basalt fibers, makes them an attractive choice for reinforcing thin-walled structures, prefabricated elements, and other components where weight is a critical factor. The application of basalt composites in 3D printing technology in construction opens new possibilities for design and construction realization. Thanks to 3D printing, it is possible to precisely place basalt fibers within the structure, optimizing their reinforcing properties and achieving optimized strength parameters while reducing material usage. Polymer Fiber-Reinforced Concrete, which is made by adding short polymer fibers to concrete, transforms the traditional building material into an advanced composite that features significantly improved performance parameters. The integration of polymer fibers, such as polypropylene or polyester, with concrete leads to a substantial improvement in the material’s flexibility and its ability to absorb energy, directly impacting increased resistance to dynamic loads and impacts. This makes fiber-reinforced concrete more resistant to various types of mechanical damage, which is particularly important for structural elements exposed to intensive operational loads [115,116]. Polymer fibers embedded in the concrete structure act as micro-reinforcements distributed throughout the material’s volume, significantly limiting the possibility of localizing and propagating microcracks. While microcracks in traditional concrete can develop and lead to more severe structural damage, the presence of polymer fibers allows for the distribution of stresses and increases material integrity. This is especially important in situations where concrete is exposed to cyclic or variable loads, which is typical for many engineering structures. Moreover, polymer fiber-reinforced concrete exhibits better resistance to the effects of atmospheric factors, including freeze–thaw cycles, which is crucial for maintaining the durability of structures in harsh climatic conditions. Polymer fibers, being resistant to corrosion, do not degrade in a humid or chemically aggressive environment, contributing to maintaining the mechanical properties of concrete over a long period. In a technological context, the use of polymer fiber-reinforced concrete does not require significant changes in the production process. Fibers can be easily added to the concrete mix during the standard mixing process, making this technology economically attractive. The possibility of using polymer fiber-reinforced concrete in both traditional construction methods and innovative techniques such as 3D printing opens new perspectives for the construction industry, allowing the realization of structures with increased durability and safety [103,114,117].

Natural Fiber Composites—the use of natural fibers in concrete composites opens new possibilities for sustainable construction, aligning with the growing trend of seeking ecological and renewable materials in the construction industry. Fibers such as flax, hemp, or sisal, with their unique properties, are used as an alternative to traditional reinforcements, introducing an element of sustainable development into modern technologies such as 3D printing. Integrating natural fibers with concrete in the 3D printing process allows for the creation of structures with improved acoustic and thermal insulation properties, which is especially valued in residential and commercial construction [118,119,120]. Additionally, natural fibers contribute to improving the plasticity of the concrete mix, facilitating the printing process and allowing for more complex architectural forms. The use of natural fiber composites also positively impacts the working environment, as these materials are less irritating and safer to use than synthetic fibers, leading to better working conditions during material production and processing [121,122]. Moreover, at the end of the construction’s life cycle, concrete with natural fibers is easier to recycle or biodegrade, contributing to reducing construction waste and its negative environmental impact. Apart from ecological and practical aspects, natural fiber composites also offer aesthetic benefits, allowing for the creation of unique textures and finishes that highlight the natural character of the material. This feature is particularly appreciated in architectural projects where there is a desire to harmoniously combine modern technologies with natural elements. However, introducing natural fibers into concrete in the context of 3D printing requires a thorough understanding of their properties and impact on the concrete mix. Factors such as water absorption by the fibers, their impact on the mix’s setting time, and interactions with other mix components must be considered. Therefore, the development of this technology requires interdisciplinary research and experiments aimed at optimizing the proportions of ingredients and printing process parameters [123,124].

### 5.5. Self-Healing Materials

**Figure 7:**
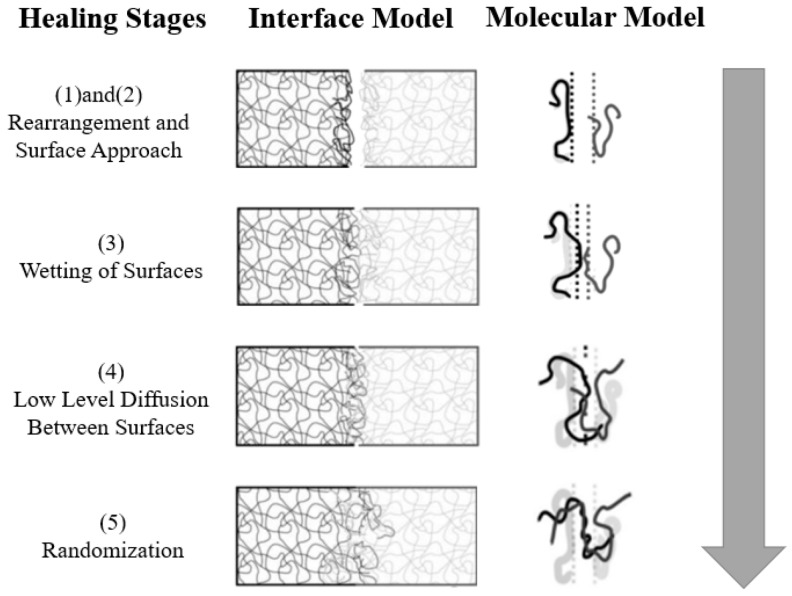
Healing mechanism of molecular interdiffusion in a fractured interface: (1) rearrangement; (2) approach; (3) wetting; (4) diffusion; and (5) randomization [128].

Self-healing materials represent a breakthrough in material engineering, opening new horizons for the durability and extended lifecycle of construction materials. These innovative mixtures, enriched with microcapsules containing self-healing substances, are designed to activate a “healing” process for microcracks that may appear in the concrete structure. When a crack compromises the integrity of a microcapsule, substances capable of reacting with each other or with concrete components are released, initiating a regeneration process that effectively “closes” the crack [125,126]. The mechanism of these materials is based on advanced polymer chemistry and other reactive substances that can be programmed to activate binding processes in response to specific conditions, such as the presence of moisture or changes in pressure within the crack. This process not only prevents further crack propagation but also restores the original mechanical strength of the material, which is crucial for maintaining structural integrity and safety [127]. The healing mechanism is presented in Figure 7.

The application of self-healing materials in 3D printing technology in construction opens new possibilities for design and construction realization. Three-dimensional printing enables the precise placement of microcapsules within the structure, maximizing the efficiency of the self-healing process and allowing for its adaptation to specific project requirements. For example, in areas with increased susceptibility to cracking, the concentration of microcapsules can be increased to provide additional protection. Introducing self-healing materials can significantly impact the economy of construction by reducing maintenance and repair costs and lowering the risk of structural failures. Additionally, by extending the lifespan of construction materials, they contribute to sustainable development by reducing the need for new raw materials and limiting construction waste. The development and implementation of self-healing materials in construction require further research and experiments aimed at optimizing the mixture’s composition, healing mechanisms, and their impact on the overall properties of the structure. Understanding the long-term effects of self-healing on the material’s behavior in various environmental and operational conditions is also essential [129,130].

## 6. Challenges and Directions of Development

Three-dimensional printing technology in construction, though promising, faces a range of technical and operational challenges that must be addressed to be fully utilized on a larger scale. Scaling 3D printing technology to produce large construction structures is one of the key challenges the construction industry must face. While 3D printing offers innovative possibilities in design and realization, existing technological and operational limitations present a barrier to its application on a larger scale, especially in the context of building complete buildings or large structural elements. Adapting existing building materials to the specific requirements of 3D printing, such as appropriate viscosity, fast drying time, and sufficient strength, is one of the key challenges in this dynamically developing field. The materials used in the printing process must allow for easy flow through 3D printer nozzles while maintaining sufficient rigidity so that the layers can retain their shape after application. This requires a complex balance of the materials’ rheological properties, which must remain fluid during extrusion and quickly transition to a solid state after application to ensure the structure’s stability during printing. Additionally, a crucial aspect is the material’s setting speed, which cannot be too slow to avoid delaying the construction process but also not too fast to allow proper bonding of subsequent layers [131]. The materials must also exhibit high strength and durability so that printed structures can match or even exceed those made using traditional methods in terms of durability and user safety. Ensuring strong adhesion between the layers is also essential for the structural integrity of printed objects, especially for large structures and complex geometries. In response to these challenges, the research and development sector is intensively working on creating new composite materials that will be optimized for 3D printing. Hybrid materials combining various components, such as cement with polymer additives or reinforcing fibers, can offer better rheological properties and accelerate the setting process while increasing the strength and durability of the final prints. The development of self-curing materials that can react to light, heat, or other external factors opens new possibilities for fast and efficient building [132]. Additionally, research into materials with self-healing properties, which can “heal” any cracks or damage, can significantly impact the longevity of printed structures. Ensuring that structures produced using 3D printing technology meet all building standards and are comparable to traditional construction methods in terms of strength and durability is a challenge that requires focused attention on many levels. It is not just about adapting the materials to the specificities of 3D printing, but also ensuring that the final structures are durable and strong. Key in this process is studying and developing materials that not only work perfectly with 3D printing technology but also possess the necessary mechanical properties, such as high compressive and tensile strength and resistance to variable weather conditions, including temperature changes, humidity, and UV radiation. Equally important is the issue of the structural integrity of printed objects [133]. The 3D printing process involves applying material layers, which means ensuring strong adhesion between the layers is crucial for the entire structure to be solid and coherent. Any heterogeneity or voids within the structure can pose a potential threat to its strength and durability. Therefore, precise control of the printing process is necessary to ensure the homogeneity and consistency of the print. However, for these materials and techniques to be widely used in construction, it is also necessary to develop and implement specific standards and regulations for 3D printing in this industry. These regulations must cover both material and procedural issues, as well as methods for testing the strength and durability of structures. Each structure produced by the 3D printing method should undergo rigorous testing to confirm its ability to withstand loads and long-term resistance to external factors. Ensuring the high precision and quality of prints in 3D printing technology is essential for constructions to be safe and durable. This challenge becomes particularly important when considering scaling the printing process for larger construction projects. There are several key aspects to focus on to achieve and maintain consistent quality throughout the printing process. Precision in 3D printing is crucial for ensuring that each layer is applied according to the design without unwanted shifts or errors that could affect the structural integrity of the printed object. This requires advanced control and monitoring systems in 3D printers, capable of precisely controlling the print head’s position and the amount and speed of the applied material. Consistent quality of the materials used is essential for ensuring print uniformity. Any changes in composition, viscosity, or other material properties can lead to differences in print quality, including adhesion issues between layers, porosity, or strength. Material suppliers must therefore ensure the high quality and consistency of their products [134]. The development of systems for automatic real-time monitoring and control of the 3D printing process plays a crucial role in ensuring the quality and consistency of printed structures, which is extremely important in the context of construction, where safety and durability are priorities. These monitoring systems use advanced sensors and cameras to continuously analyze each layer during the printing process, allowing for the immediate detection and correction of any irregularities such as voids, layer shifts, or material unevenness. By utilizing technologies based on artificial intelligence and machine learning, these systems can be trained to recognize error patterns and automatically correct them, allowing for dynamic adjustments to the printing process based on continuous learning from previous prints. Artificial intelligence (AI) and machine learning (ML) technologies are significantly contributing to improving the quality, precision, and uniformity of 3D-printed structures in the field of additive manufacturing. By analyzing massive amounts of data from printing processes, AI and ML algorithms optimize printing parameters such as speed, temperature, and layer thickness, leading to continuous process improvement [135]. These systems also monitor printing in real-time using sensors and cameras, identifying and correcting errors immediately [136]. In addition, ML-based predictive models analyze historical data, predicting defects in printed structures and enabling preventive parameter adjustments, significantly improving the quality of the final product [137]. The integration of AI and ML in the 3D printing process leads to more efficient, precise, and reliable additive manufacturing. Integration with CAD/CAM software enables detailed planning of the printing process and automatic corrections directly in the digital model, significantly increasing print precision and efficiency. The integration of CAD and CAM software significantly improves the precision and efficiency of 3D printing in the construction industry, enabling the creation of detailed and accurate 3D models that are directly converted into instructions for 3D printers. With CAD, designers can accurately specify geometries and tolerances, minimizing design errors, while CAM optimizes manufacturing processes by automating toolpaths, increasing efficiency, and reducing production time and costs. Studies have shown that this integration leads to a significant increase in the quality and uniformity of printed parts, which is crucial in construction, where precision and durability of structures are priorities [138,139]. Systematic monitoring of each layer ensures the consistency of the entire object and allows for immediate response to any irregularities, which is crucial for maintaining the high quality and durability of printed structures. Adaptive printing, which adjusts printing parameters on the fly in response to changes in materials, ambient conditions, or other external factors, ensures process optimization and minimizes the risk of errors. In addition to real-time monitoring, it is also essential to conduct strength tests and validation of finished prints to ensure they meet all required standards. Implementing advanced monitoring and control systems in the 3D printing process in construction is key to ensuring that printed structures are safe, durable, and compliant with designed specifications, opening new possibilities for construction and allowing for more sustainable and efficient construction practices. The effective integration of 3D printing with traditional construction methods is a key element in fully utilizing the potential of this innovative technology in the construction sector. This process requires significant changes in the approach to designing and planning construction projects, as well as a commitment to developing skills and knowledge among industry workers. First and foremost, integrating 3D printing requires adapting design processes. Architects and engineers must consider the specific possibilities and limitations of 3D printing as early as the conceptual stage, which means thinking about structures in a way that allows for their efficient printing. This approach may require the use of new design tools, such as advanced CAD software capable of precisely modeling objects for 3D printing and simulating construction processes. These changes also have a direct impact on construction planning. Traditional methods must be adapted to consider the specificities of the printing process, such as the time needed to print individual elements, the logistics of delivering printing materials to the construction site, and the coordination between printing stages and conventional construction work. Planning must also consider potential limitations related to the size of 3D printers and their mobility, which can affect the strategy for implementing large projects. In addition to technical and organizational aspects, a crucial factor for success is training construction workers in new technologies. Workers must understand not only how to operate 3D printers and related equipment but also how 3D-printed materials interact with traditional construction materials and how they can be used to create durable, safe structures [51,57]. This training should include both theoretical and practical aspects, allowing workers to acquire the skills necessary for effectively utilizing 3D printing on construction sites. Implementing these changes requires a holistic approach that combines design innovations, strategic planning, and skills development. Only through such integrated actions can 3D printing become a full-fledged complement to traditional construction methods, opening new possibilities for the construction industry and contributing to its further development towards greater efficiency, sustainability, and innovation. Assessing the environmental impact of 3D printing is essential to understand and maximize the ecological benefits that this technology can bring. In terms of energy consumption, 3D printing has the potential to be more energy efficient than traditional production methods, primarily due to its principle of “adding” material only where needed, minimizing waste. However, the actual energy efficiency of the 3D printing process can vary significantly depending on the type of printer, the material used, and the complexity of the printed object. For example, 3D printing processes that require high temperatures to melt the material may consume more energy. Therefore, it is important to develop and implement strategies to optimize energy consumption in 3D printers, such as improving thermal insulation or using more efficient heating systems. Regarding waste generation, 3D printing offers significant advantages over traditional production methods. By adding material only in required places, 3D printing minimizes the amount of waste produced in the process of creating objects. Moreover, the possibility of using recycled materials to produce filaments for 3D printers opens the way for even more sustainable production practices. Nevertheless, it is crucial to further study and develop printed materials to increase their environmental friendliness, for example, by seeking biodegradable or more easily recyclable raw materials. Developing more sustainable 3D printing practices also requires a holistic approach to the entire lifecycle of 3D-printed products, from design through to production, use, and disposal. Designing with ecology in mind (eco-design) can significantly contribute to reducing the ecological footprint of 3D-printed products, for example, by optimizing their geometry to minimize material and energy consumption during production and increasing product efficiency and durability. Additionally, incorporating the principles of a circular economy, where materials at the end of a product’s life are reused or recycled, can significantly contribute to the sustainable development of 3D printing technology. In the context of sustainable development, it is also crucial to engage stakeholders from various sectors—from equipment and printing material manufacturers to technology users, regulatory bodies, and research institutions—to collaborate on creating standards and norms that support ecological practices in 3D printing. Only through integrated actions can 3D printing achieve its full potential as a technology contributing to sustainable development [134,140]. Also of interest is the impact of 3D technology on the environment. Shuaib et al. studied the relationship between this technology and the environment. Among their observations, they stressed that 3D printing is a better alternative for manufacturing complex and low-volume parts. They observed that the environmental impact of 3DP is 70% less compared to the traditional manufacturing process. The advantages and disadvantages of implementing different 3D technologies in terms of their on the environment are presented in Table 7 [141].

An important aspect is the scaling of the 3D printing process for larger construction projects, which involves a number of significant challenges. These challenges affect the efficiency, quality, and cost-effectiveness of implementing such projects. A key challenge is to ensure that the printing equipment is scaled appropriately, as most available 3D printers are tailored for small or medium-sized structures, making them difficult to use for large buildings or infrastructure complexes. In addition, the 3D printing process requires precise material management, and in large projects, difficulties related to material consistency and properties can lead to problems with the quality and durability of structures. The logistical challenges of delivering and stacking large quantities of materials and managing the length of the printing cycle are other difficulties that can affect project schedules and costs. Another major issue is integrating 3D printing with existing construction methods and ensuring compliance with construction and quality standards, which often require adjustments to regulations and procedures. Finally, the costs associated with investing in large and sophisticated 3D printing technology, including advanced machinery, high-performance materials, and appropriate personnel training, can be significant, affecting the economics of the overall process. Research into these challenges points to the need for further development of 3D printing technology, optimization of materials, and adaptation of construction methodologies to enable efficient and scalable application of this technology in large-scale construction projects.

## 7. Summary and Conclusions

Three-dimensional printing technology in construction presents groundbreaking potential in the context of innovative design, rapid realization, and sustainable development. Introducing this technology into the construction sector opens possibilities for creating complex geometries, reducing waste, and shortening construction times. By using various materials, from traditional concrete mixtures through to geopolymers and advanced composites with natural and polymer fibers, 3D printing adapts to a wide range of applications in construction. To fully utilize the potential of 3D printing, it is necessary to further study and develop building materials optimized for printing processes. This requires focusing on rheological properties, setting speed, and material durability to ensure the high quality and safety of structures. Ensuring high precision and print quality is crucial for the durability and safety of structures. This requires advanced monitoring and control systems that enable continuous assessment and correction of the printing process in real-time. Effective integration of 3D printing with traditional construction methods is essential to fully utilize this technology. This requires adapting design processes, construction planning, and training workers in new technologies. Three-dimensional printing offers significant environmental benefits by reducing waste and introducing the possibility of using recycled materials. To maximize these benefits, it is necessary to adopt a holistic approach to managing the entire lifecycle of 3D-printed products, from design to disposal. Engaging stakeholders from various sectors in creating norms and standards supporting ecological and efficient practices in 3D printing is crucial for the sustainable development of this technology.

In summary, 3D printing in construction presents a promising direction for development, which can bring significant benefits both for the industry and the environment. However, to fully utilize its potential, further research, technological development, and cooperation between various disciplines and sectors are necessary.

## Figures and Tables

**Table 1:** Various 3D Printing Technologies and Their Applications.

3D Printing Technology	Material Used	Key Applications	Advantages	Disadvantages
Fused Deposition Modeling (FDM)	Thermoplastics	Prototypes, structural elements	Low cost, variety of materials	Lower strength, less precision
Selective Laser Sintering (SLS)	Powdered materials	High-strength components	High strength, complex shapes	High cost, slower process
Stereolithography (SLA)	Photopolymer resins	Architectural models, decorative elements	High precision, surface quality	Limited material choices, post-processing needed

**Table 2:** Properties and Requirements of High-Performance Building Mixtures.

Property	Requirement
High compressive strength	Withstand significant loads
Durability	Resist moisture, temperature changes, chemicals, and corrosion
Workability	Easy mixing, transporting, placing, and compacting
Sustainability	Use of recycled materials, reduced CO_2_ emissions

**Table 3:** Selected properties of concrete mixtures.

Property	Description	Example value	References
Composition	Types and proportions of materials used in the concrete mix	Cement, sand, water, additives	[64,65,66]
Compressive strength	The maximum stress that the mixture can withstand before failure	20–50 MPa	[67,68,69,70]
Open time	Open time is the period at which the material can be extruded continuously without separation	15–90 min	[65,71,72]
Plastic shrinkage	Reduction in the volume of fresh concrete mix during drying out	Depends on the additives used, mostly less than 2%	[73,74,75]

**Table 4:** Comparisons between geocement and Portland cement based on their eco-friendliness and sustainability [88].

Criteria	Geocement	Portland Cement
CO_2_ emissions	Low to none	Extremely high
Sustainability	High	Low
Energy saving	High with no embodied energy	Low with greater embodied energy
Costs (production, sales, etc.)	Low	Extremely high
Eco-friendliness	High	Low
Water requirement	Low	High
Availability of raw materials	Abundant and cheap	Non-abundant and costly
Thermal conductivity	Low	High
Ability to adsorb and immobilize toxic substances	High	Moderate to high
Preparation technique	Simple	Complex
Volume stability	Good	Fair
Setting time	Short (about 10–60 min)	Long (about 30–300 min)
Global warming contribution	Low to none	High

**Table 5:** Advantages and Disadvantages of Using Different Types of Fibers.

Type of Fiber	Advantages	Disadvantages
Glass Fibers	- High tensile strength and chemical resistance - Lightweight - Improved thermal insulation	- Brittleness - Requires special additives to prevent alkali reaction - Can cause skin irritation
Carbon Fibers	- Very high tensile strength and low weight - Corrosion resistance - Excellent dimensional stability	- High cost - Brittleness Difficult to recycle
Basalt Fibers	- High tensile strength and resistance to high temperatures - Chemical and biological resistance - Good adhesion to the cement matrix	- Higher cost compared to glass fibers - Heavier than carbon and glass fibers - Limited availability on the market
Natural Fibers	- Environmental friendliness - Low weight - Good flexibility - Low cost	- Variability in properties depending on the source - Potential for biodegradation - Lower durability compared to synthetic fibers - Sensitivity to moisture

**Table 6:** Physical Properties of BFs [112].

Parameter	Value
Specific gravity	2.65
Water Absorption (%)	3.60
Density (g/cm^3^)	2.64–2.75
Tensile Strength (MPa)	2800–4800
Elastic modulus (GPa)	89–110
Elongation (%)	3.05–4.00

**Table 7:** Deploying 3D printing technologies: positive and negative impacts on the environment [141].

Process	Positive Impact	Negative Impact
EBM, SLS, SLM	Design can be improved topologically for better performance and to reduce total energy. The material can be recycled. Process residues are negligible.	Extra energy spent on the preparation of raw powdered material. Resource consumption in the form of gases such as compressed air, argon, and nitrogen increases cost. Disposal consumes a great deal of energy. It contributes heavily to freshwater, marine, and terrestrial ecotoxicity.
SLA	Energy spent in post-processing is almost negligible. Process residues are negligible.	It has the highest material preparation energy indicator, i.e., a high specific energy consumption. Energy is wasted in preheating the equipment.
FDM	Resource consumption is negligible. The material can be recycled. Process residues are negligible.	The total energy indicator is the highest in FDM processes as it consumes a large amount of energy when heating the material. It contributes to freshwater and marine eutrophication.

## Data Availability

Not applicable.
